# Individual Peculiarities of the Male Urethra

**Published:** 1904-10

**Authors:** W. B. Emery

**Affiliations:** Atlanta, Ga.


### INDIVIDUAL PECULIARITIES OF THE MALE URETHRA.

BY W. B. EMERY, M.D.

ATLANTA, GA.

In order to introduce this most commonplace subject, I deem it necessary to refresh our minds concerning the most frequent affections to which the urethra is subject. First, strictures, which may be classified as inflammatory, organic and spasmodic. Second, the infections, which are in large part gonorrheal or simple. Of course, there are other affections, which involve the urethra, such as chancre, chancroid, abscess, fistula and congenital malformation; but strictures and urethritis form so large a part of the office work of genito-urinary surgeons, that it is concerning the treatment of only these conditions I desire to call your attention.

None of you, who have treated the urethra in its various pathological conditions, can fail to be impressed with the fact, that the subject I shall attempt to discuss is a timely one. I know of few conditions which are more abused by the laity than these urethral affections. There is a habit of passing from friend to friend, the formula for an injection which was originally prescribed for a specific case. The drug.stores are full of certain nostrums or patent medicines for urethral discharges, regardless of their cause, and quacks feed and grow fat on this class of cases. Furthermore, I am constrained to say, that from what I have heard there is a tendency to carelessness, even among some of the best men of our profession, when it comes to the diagnosis and treatment of these cases. How often have I heard the questions asked: "What is your prescription for gonorrhea, or how do you treat a stricture?" These questions can never be intelligently answered, for there are rarely two patients which should be treated exactly alike. You may start two cases of gonorrhea on the same prescription for injection and find after the first treatment it will be necessary to reduce the strength of the solution for one, perhaps, lengthen the period between the injections, probably reduce the pressure, and possibly be forced to change to an entirely different prescription.

You may have two cases of stricture which on examination you find are of the same calibre and situated at practically corresponding portions of the urethra. You begin the treatment—the same in both cases—and find in a short time that it will be necessary to change the manner, and sometimes the method, in one of the cases. For example, you find that one will admit of the passage of sound much oftener than the other; that one stricture will yield and become absorbed very rapidly, while the other will become inflamed and discharge, and you are able to increase the size of sound very slowly, or perhaps discard the use of steel sound altogether—resort to irrigation for a time, and then use the rubber bougie. You may find even then that you make no progress, and be forced to cut the stricture. These cases, remember, presented the same symptoms at the beginning.

With the patient standing, I use the hydrostatic pressure with greatest satisfaction in many cases, but with some I am forced to use the hand syringe with patient in recumbent position. I have had patients almost faint from the use of hydrostatic pressure with mild permanganate solution, while others experience a sense of relief and pleasure, with strong solution of the same drug. I have, more than once, seen the passage of a steel sound one time cause the testicles to swell, while in other cases the frequent passage of these instruments for a number of months will not cause the least irritation or pain in these organs.

Such being the case, there are a few points we should remember in beginning the treatment of a urethra, namely: Remember not only its anatomy, but its histological and physiological character; that it is lined with a most sensitive and delicate mucous membrane; begin the treatment of each case guardedly.

For urethritis, begin with mild solutions and slight pressure. In examining for stricture be gentle. There are many urethras which will apparently bear much punishment at the time you examine, but unless nature works wonders the patient will reap the results of laceration later. Some men seem to think that they have not done the patient justice unless the urethra bleeds during or after the examination.

Become as thoroughly acquainted with each urethra as possible before you enter upon a certain line of treatment.

The whole purpose of these remarks is to remind us that certain individual peculiarities do exist in these organs, and prompt us to be on our guard that we may meet them in the proper manner.

*1103 Empire Building.*

---

AN exchange says none of the more striking experiments performed with the new *n*-rays are more wonderful than the recent investigation by Becquerel, in which he has ascertained that certain inorganic bodies, such as calcium sulphide, which emit these rays after having been exposed to sunlight, when subjected to the influence of an anesthetic, such as chloroform, ether or nitrous oxide, lose this power. A few months ago Meyer found that this would happen with plants, but that an anesthetic should affect an inorganic substance is an entirely different matter, and brings up a new and difficult question for scientists to answer. It may even mean the breaking down of the line between organic and inorganic substances, and that the *n*-rays are the result of certain vital processes which anesthetics may check. In other experiments of a similar nature it has been found that the variations of the *n*-rays produced by the nervous centers, as indicated by the glowing of the phosphorescent screen on which they were received, enable an observer to follow the action of an anesthetic and to determine when danger to life begins, since it is possible to distinguish readily the point of death. The development of this experiment will doubtless afford an interesting method of studying anesthesia, and it may be possible to use it to indicate the time necessary for employing restorative measures when a patient is under the influence of an anesthetic. Such a use in the light of present experiments is the barest possibility, but it indicates a field that may be opened up by this new discovery.

